# Ameliorative Effect of Chitosan/*Spirulina platensis* Ethanolic Extract Nanoformulation against Cyclophosphamide-Induced Ovarian Toxicity: Role of PPAR-γ/Nrf-2/HO-1 and NF-kB/TNF-α Signaling Pathways

**DOI:** 10.3390/md22090395

**Published:** 2024-08-30

**Authors:** May Almukainzi, Thanaa A. El-Masry, Hanaa A. Ibrahim, Hebatallah M. Saad, Enas I. El Zahaby, Asmaa Saleh, Maysa M. F. El-Nagar

**Affiliations:** 1 Department of Pharmaceutical Sciences, College of Pharmacy, Princess Nourah bint Abdulrahman University, P.O. Box 84428, Riyadh 11671, Saudi Arabia; mkalmukainizi@pnu.edu.sa (M.A.); asali@pnu.edu.sa (A.S.); 2 Department of Pharmacology and Toxicology, Faculty of Pharmacy, Tanta University, Tanta 31527, Egypt; thanaa.elmasri@pharm.tanta.edu.eg (T.A.E.-M.); hanaa.abdelkareem@pharm.tanta.edu.eg (H.A.I.); 3 Department of Pathology, Faculty of Veterinary Medicine, Matrouh University, Cairo 51511, Egypt; heba.magdy@mau.edu.eg; 4 Department of Pharmaceutics, Faculty of Pharmacy, Delta University for Science and Technology, Gamasa 35712, Egypt; enas.elzahabi@deltauniv.edu.eg

**Keywords:** cyclophosphamide, chitosan/*Spirulina platensis*, ethanolic extract, ovarian toxicity, Nrf-2

## Abstract

Cyclophosphamide (CP) is an anticancer drug that causes infertility disorders. This study was designed to evaluate a nanoformulation of chitosan with an ethanolic extract from *Spirulina platensis* in terms of its protection against cyclophosphamide-induced ovarian toxicity. Nine groups of female Wistar rats were randomly assigned as follows: 1: control vehicle, 2: chitosan polymer, 3: telmisartan, 4: *Spirulina platensis* extract, 5: nanoformulation of the *Spirulina platensis*, and 6: single injection of CP; groups 7, 8, and 9 received the same treatments as those used in groups 3, 4, and 5, respectively, with a single dose of CP (200 mg/kg, I.P). The results displayed that the CP treatment decreased estradiol, progesterone, anti-mullerian hormone, and GSH content, and it downregulated PPAR-γ, Nrf-2, and HO-1 gene expression. In addition, the CP treatment caused an increase in the FSH, LH, and MDA levels. In the same manner, the protein expression of caspase-3, NF-kB, and TNF-α was upregulated in response to the CP treatment, while PPAR-γ was downregulated in comparison with the control. The rats treated with SPNPs exhibited a substantial reduction in the detrimental effects of oxidative stress and inflammation of the ovarian tissue. This study’s conclusions showed that SPNPs counteracted the effects of CP, preventing the death of ovarian follicles and restoring the gonadotropin hormone balance and normal ovarian histological appearance.

## 1. Introduction

A common alkylating chemotherapeutic drug used to treat a variety of cancer forms is cyclophosphamide [1]. The drug has remarkable efficacy when utilized either alone or in combination with additional chemotherapeutics, even with its cytotoxic [2], hepatic [3], renal [4], and neurological side effects and adverse consequences [5]. Cyclophosphamide has been used in clinical settings for over 50 years [6]. Chemotherapy-induced gonadotoxicity is now a recurring cause of premature ovarian insufficiency (POI) due to the rising incidence of cancer among young women [7]. Cyclophosphamide is destructive to the reproductive organs of women—particularly the ovary [8]. The metabolites of cyclophosphamide bind to DNA and inhibit DNA synthesis, which is responsible for the drug’s toxic effects [9]. In addition, it forms reactive oxygen species (ROSs) when conjugated with glutathione, compromising the ovary’s antioxidant defense mechanism [8]. Furthermore, after cyclophosphamide therapy, malondialdehyde (MDA), a marker of lipid peroxidation, increases [8].

Antioxidants are now being studied as a potential treatment for the side effects of cyclophosphamide [10]. Antioxidants have been shown to have protective properties against the damage caused by cyclophosphamide in numerous studies [11]. Cyclophosphamide-related biochemical and physiological problems are reduced when antioxidant chemicals are used [12].

Initially, a blue-green microalga from the *Oscillatoriaceae* family, *Spirulina platensis* (*Arthrospira platensis*), was utilized as a source of vitamins and protein [13]. Additionally, this microalga has a high content of phycocyanin, beta-carotene, B12, and phycocyanobilin [14]. Moreover, selenium (Se), essential amino acids, vitamins (A and E), and fatty acids (linoleic acid and palmitic acid) are abundant in *Spirulina platensis*. Medical researchers have been interested in *Spirulina* because of its unique qualities, which include anti-inflammatory, antiviral, anticancer, antiapoptotic, antioxidant, and immunomodulatory properties [15,16,17]. In recent years, there have been numerous studies on spirulina’s protective capacity, as it enhances the activity of antioxidant enzymes. This, in turn, helps reduce the levels of free radicals, thereby preventing lipid peroxidation and DNA damage [17,18]. 

Chitosan is an environmentally friendly polysaccharide that breaks down spontaneously. Chitosan primarily exerts its biological effects on the major amine group located on the carbon of the glucosamine residue [19]. Chitosan’s biological activity is directly proportional to its molecular weight and degree of deacetylation; the greater the biological effect, the lower the molecular weight and the higher the degree of deacetylation [20].

Furthermore, internal chitosan features include mucoadhesion, regulated drug administration, and increased permeability. For these qualities, both hydroxyl and amino groups are necessary [21]. High-encapsulation-efficiency micro- and nanoparticles for medical and biological applications may be synthesized using the straightforward, adaptable ionotropic gelation process. This method has been effectively used to encapsulate several medications, including interferon and doxorubicin [22]. The process of complex coacervation takes place when two biopolymers with opposite charges interact in an aqueous colloidal medium, resulting in the separation of a solution into two distinct liquid phases [23].

It has been discovered that a high degree of deacetylation in chitosan, coupled with a specific molecular weight range, is essential for controlling the particle size distribution during the fabrication of chitosan nanoparticles through the process of ionic gelation using chitosan and TPP (tripolyphosphate). TPP is commonly favored as the cross-linking agent for chitosan nanoparticles due to its multivalency, non-toxic nature, and ability to form gels through ionic interactions [22,23]. The behavior of NPs in biological fluid, as well as their intracellular absorption and trafficking, is significantly influenced by their size. It also affects these nanoparticles’ stability, drug loading, release, toxicity, and in vivo biodistribution [24].

Furthermore, the activation of the angiotensin II type 1 (AT1) receptor by angiotensin II, the principal active component of the renin-angiotensin system, triggers oxidative stress and inflammation [25]. The selective AT1-receptor blocker telmisartan revealed major antioxidant and anti-inflammatory properties [26,27,28]. Telmisartan was utilized as a standard drug to evaluate the protective properties of *Spirulina platensis* extract and a nanoformulation of chitosan/*Spirulina platensis*.

Therefore, this study sought to assess the potential protective benefits of *Spirulina platensis* or a nanoformulation of chitosan/*Spirulina platensis* while clarifying the underlying mechanisms in an experimental model of ovarian toxicity in rats following the finding that cyclophosphamide may induce ovarian toxicity.

## 2. Results

### 2.1. In Vitro Analysis

#### 2.1.1. Antioxidant, Total Phenolic, and Total Flavonoid Contents of the SP Ethanolic Extract

The results in Table 1 demonstrate that the SP ethanolic extract exhibited a higher inhibition percentage of 79.7% in radical scavenging activity according to the DPPH assay, with an estimated IC_50_ value of 30.03 μg/mL. Additionally, the total phenolic content of the SP ethanolic extract was determined to be 35.9 mg GAE/g DW (Table 1). Furthermore, the content of flavonoids in the SP ethanolic extract was found to be 81.17 mg QuE/g DW (Table 1).

#### 2.1.2. Analyses of Phenolic and Flavonoid Compounds in the SP Ethanolic Extract Using HPLC

**Figure 1:**
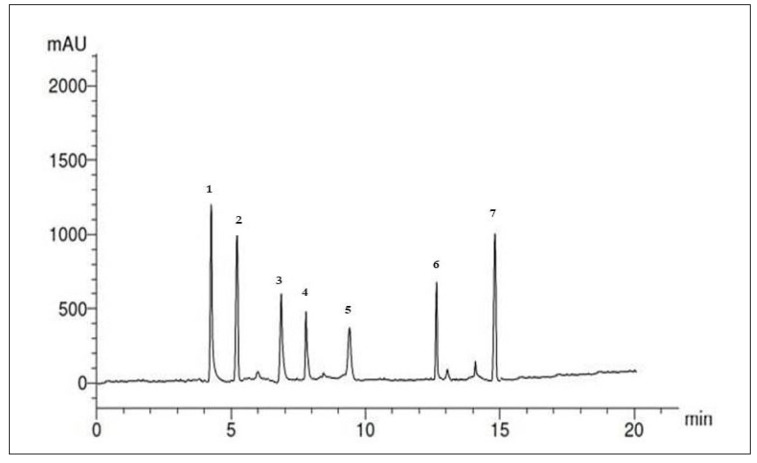
HPLC chromatogram of phenolic compounds in the SP ethanolic extract.

The profile of the SP ethanolic extract is illustrated in Figure 1, and Table 2 reveals the separation of five compounds under the specified conditions. The concentrations of phenolic compounds (µg/mL) in the SP ethanolic extract could be arranged in descending order as follows: syringgenic (12.89 µg/mL), catechol (10.31 µg/mL), cinnamic acid (3.78 µg/mL), caffeic acid (1.41 µg/mL), and pyrogallol (1.39 µg/mL).

**Figure 2:**
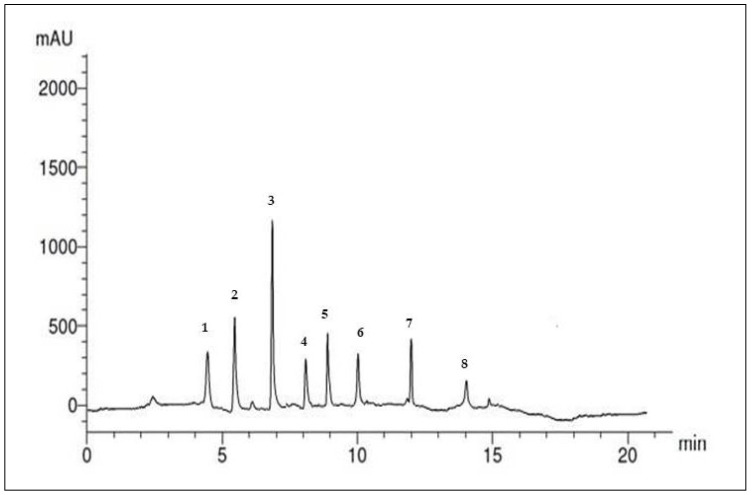
HPLC chromatogram of the flavonoid compounds in the SP ethanolic extract.

Similarly, the flavonoid compounds in the SP ethanolic extract were also identified. Figure 2 and Table 3 present the separation of seven compounds under the specified conditions. The concentrations of flavonoid compounds (µg/mL) in the SP ethanolic extract could be arranged in descending order as follows: quercetin (15.30 µg/mL), luteolin (6.41 µg/mL), rutin (6.20 µg/mL), catechin (5.26 µg/mL), kaempferol (3.36 µg/mL), apigenin (3.51 µg/mL), and naringin (3.12 µg/mL).

#### 2.1.3. GC-MS Analysis of the SP Ethanolic Extract

**Figure 3:**
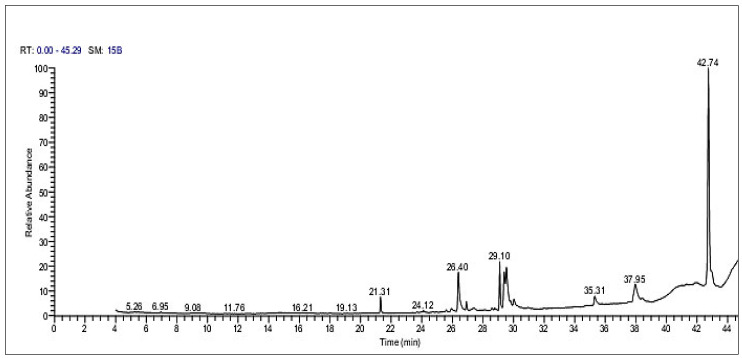
GC-MS chromatogram of the SP ethanolic extract.

As shown in Figure 3, a variety of compounds exhibiting numerous biological activities were present. These compounds primarily included hexadecanoic acid–octadecyl ester (47.92%), 9,12-octadecadienoyl chloride (Z, Z) (9.45%), 9,12-octadecadienoic acid (Z, Z) (9.11%), and phytol (8.67%). Other compounds are also listed in the table. Furthermore, the bioactive properties of all of these compounds are also listed in Table 4.

#### 2.1.4. Characterization of SP Nanoparticles

##### Analyses of the Percentage Yield, Particle Size, and Zeta Potential

**Figure 4:**
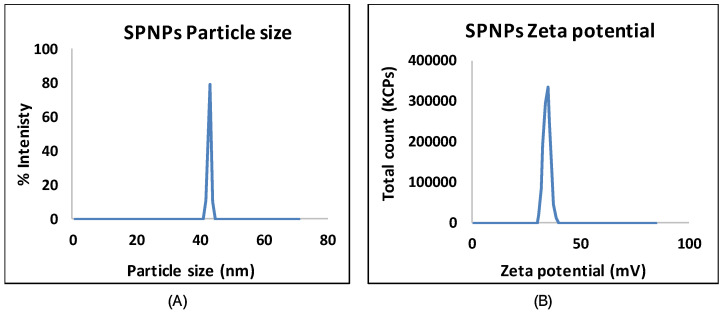
Particle size distribution (**A**) and zeta potential (**B**) of the SPNPs.

The percentage yield was 20.96 ± 2.89% (Table 5). SPNPs were evaluated using MADLS; the particle size was less than 150 nm (Figure 4A), with a mean value of 138.2 ± 7.18 nm. The zeta potential ranged from −28.33 to −33.69 mV, with an average of −31.58 ± 2.86 mV (Figure 4B).

##### Scanning Electron Microscopy (SEM) 

**Figure 5:**
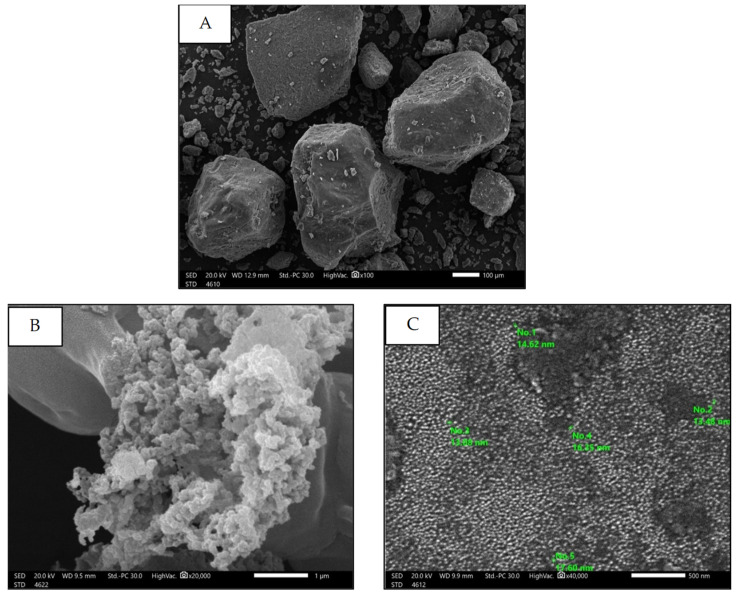
SEM of Free SP particles (**A**) and SPNPs (**B**,**C**).

SEM was used to assess the particle morphology, size, and shape of free SP and SPNPs, as illustrated in Figure 5A (image magnification: 100×). The free SP particles exhibited an irregular arrangement. In Figure 5B (image magnification: 20,000×), the SPNPs appeared to be spherical, cauliflower-like aggregates with diameters of less than 20 nm, as shown in Figure 5C (image magnification: 40,000×).

##### Thermal Stability (DSC)

**Figure 6:**
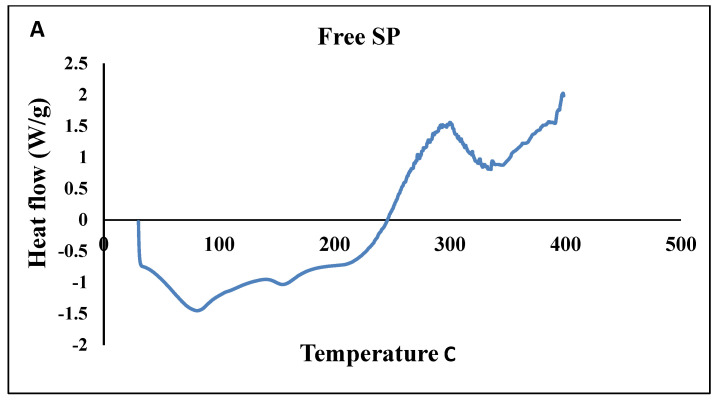

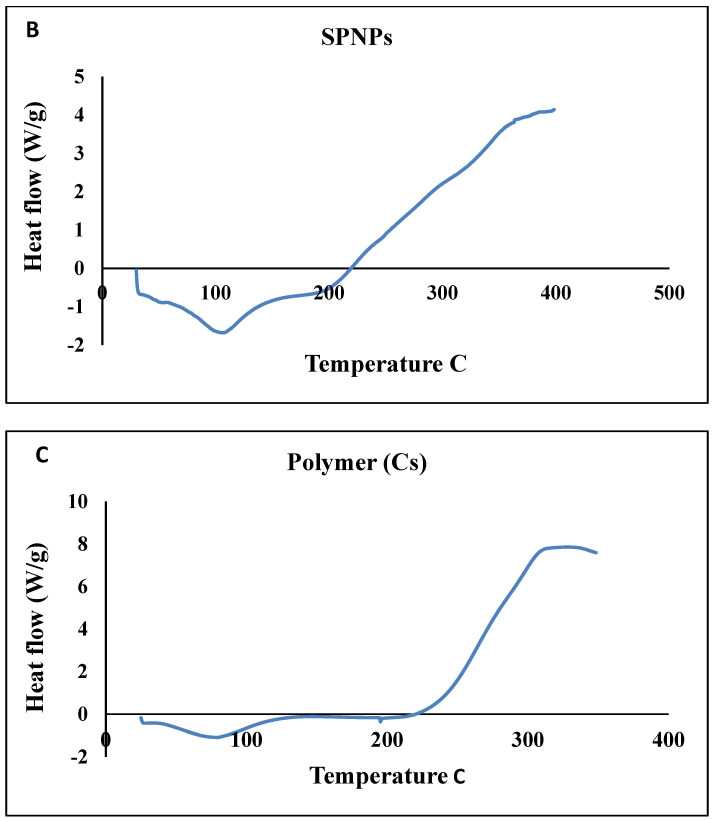
DSC analysis of Free SP particles (**A**), SPNPs (**B**), and a polymer (Cs) (**C**).

DSC analysis was conducted on free SP particles, polymer (Cs), and SPNPs, as shown in Figure 6 and Table 6. The DSC thermogram of the free SP particles (Figure 6) revealed a broad endothermic peak at 87.08 °C (−1.399 W/g) and a broad exothermic peak at 303.1 °C (1.54 W/g). The polymer (Cs) exhibited a broad endothermic peak at 78.33 °C (−1.078 W/g) and an additional exothermic peak at 328 °C (7.85 W/g). Conversely, SPNPs displayed a slight shift in the broad endothermic peak to 106.53 °C (−1.677), while the exothermic peak shifted to a higher temperature of 398.4 °C (4.13 W/g) (Figure 6 and Table 6).

##### X-ray Diffraction Analysis (XRD) 

**Figure 7:**
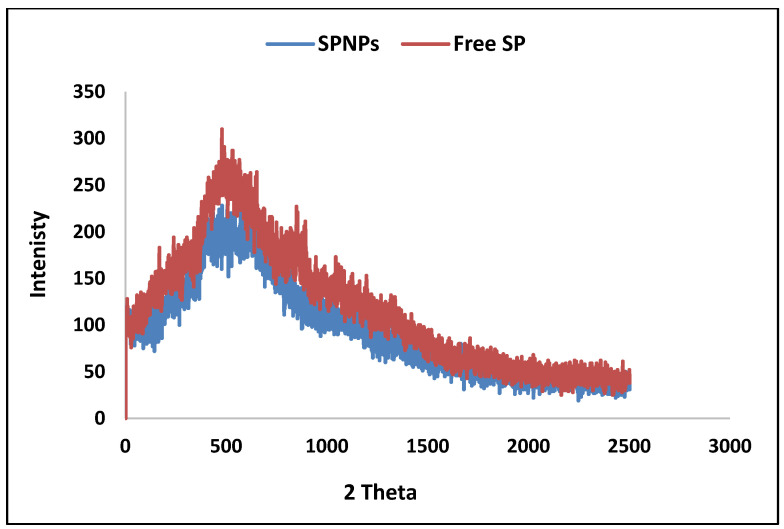
XRD analysis of Free SP particles and SPNP.

As depicted in Figure 7, the diffractograms of the free SP particles and SPNPs exhibited nearly identical patterns.

### 2.2. Biological Investigations

#### 2.2.1. Effects of Free SP Extract and the Nanoformulation on Estradiol, Progesterone, and Anti-Mullerian Hormone Levels in Serum

**Figure 8:**
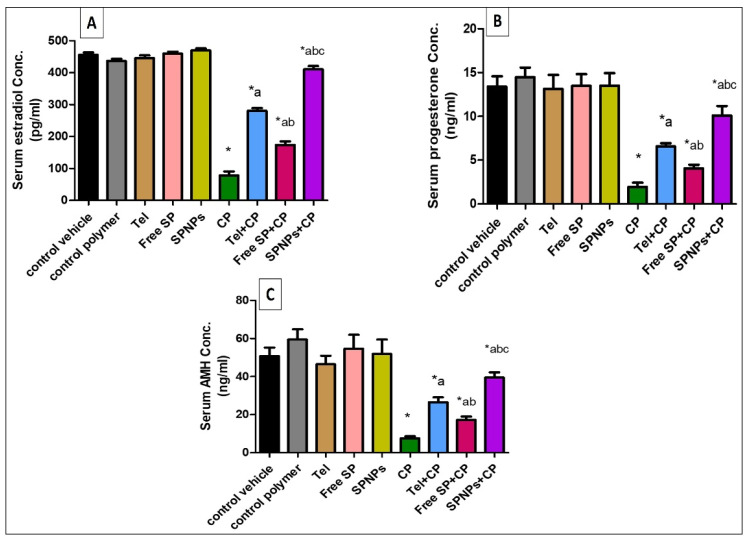
Effects of free SP extract and the nanoformulation on serum levels of estradiol, progesterone, and AMH. (**A**) Estradiol concentration; (**B**) progesterone concentration; (**C**) anti-mullerian hormone concentration. Data are presented as the mean ± SD (n = 6). Each group differed significantly from the others at a *p*-value ≤ 0.05. * versus the control group, ^a^ versus the CP group, ^b^ versus the Tel+CP group, and ^c^ versus the Free SP+CP group. CP: Cyclophosphamide, SP: *Spirulina platenesis* extract, SPNPs: *Spirulina platenesis* extract nanoformulation.

As shown in Figure 8, the cyclophosphamide group showed a marked decline (82.77%, 85.5%, and 85.2%, respectively) in levels of estradiol, progesterone, and AMH relative to the control group. On the other hand, pretreatment of rats with Tel showed a substantial increase (256%, 239%, and 252%) in serum hormone levels, pretreatment of rats with free SP extract showed a substantial increase (121.13%, 109.9%, and 130%) in hormone levels, and pretreatment of rats with SPNPs showed a remarkable increase (422.8%, 421.4%, and 426%) in hormone levels in comparison with the CP group. Moreover, rats pretreated with SPNPs showed a considerable rise (46.4%, 53.8%, and 49.07%) in hormone levels in comparison with those treated with Tel and (136.3%, 148.3%, and 128.6%) those treated with free SP extract (*p* < 0.05).

#### 2.2.2. Effects of Free SP Extract and the Nanoformulation on FSH and LH Levels in Serum

**Figure 9:**
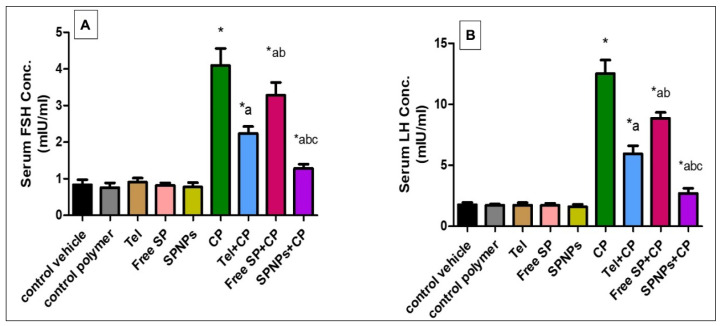
Effect of free and nanoformulation of SP on serum levels of FSH and LH. (**A**) FSH concentration (**B**) LH concentration. Data presented as mean ± SD (n = 6). Each group differed significantly from the others at *p*-value ≤ 0.05. * versus the control group, ^a^ versus the CP group, ^b^ versus the Tel+CP group, and ^c^ versus the Free SP+CP group. CP: Cyclophosphamide, SP: *Spirulina platenesis* extract, SPNPs: *Spirulina platenesis* extract nanoformulation.

As shown in Figure 9, the cyclophosphamide group showed a remarkable increase (390% and 607.3%) in the levels of FSH and LH relative to the control group. In contrast, pretreatment of rats with Tel showed a considerable decline (45.33% and 52.66%) in serum hormone levels, there was a marked reduction (19.8% and 29.27%) in hormone levels in rats pretreated with free SP extract, and there was a substantial decrease (68.7% and 78.5%) in hormone levels in rats pretreated with SPNPs in comparison with the CP group. Moreover, rats pretreated with SPNPs showed a substantial decline (42.73% and 54.56%) in hormone levels in comparison with the Tel group and (60.97% and 69.6%) in the free SP group (*p* < 0.05).

#### 2.2.3. Effect of Free SP Extract and the Nanoformulation on Oxidative Stress Biomarkers in Ovarian Tissues

**Figure 10:**
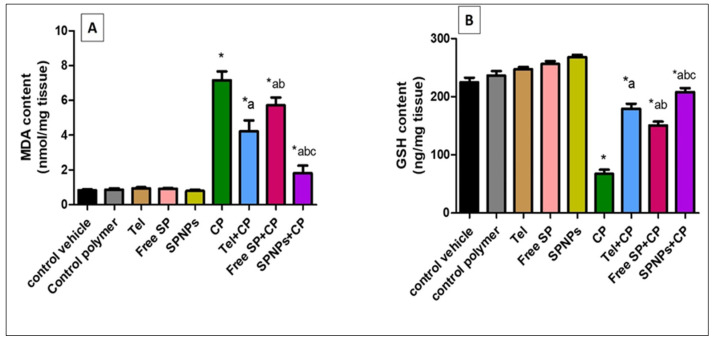
Effects of free SP extract and the nanoformulation on oxidative stress biomarkers in ovarian tissues. (**A**) MDA content; (**B**) GSH content. Data are expressed as the mean ± SD (n = 6/group). Each group differed significantly from the others at a *p*-value ≤ 0.05. * versus the control group, ^a^ versus the CP group, ^b^ versus the Tel+CP group, and ^c^ versus the Free SP+CP group. CP: Cyclophosphamide, SP: *Spirulina platenesis* extract, SPNPs: *Spirulina platenesis* extract nanoformulation.

Figure 10 demonstrates that the cyclophosphamide group showed a remarkable rise (761.4%) in MDA content in comparison with the control group. On the other hand, pretreatment of rats with Tel, free SP extract, and SPNPs resulted in a substantial reduction (40.99%, 20.1%, and 74.69%) in MDA content in comparison with the CP group. Moreover, rats pretreated with SPNPs showed a marked decline (56.93% and 68.35%, respectively) in MDA content in comparison with the Tel and free SP groups (*p* < 0.05).

In contrast, the GSH content in the CP group was considerably reduced (70%) in comparison with the control group. However, the depleted GSH content was restored by pre-treatment with Tel, free SP extract, and SPNPs by approximately 165.85%, 123.32%, and 208.38%, respectively, in comparison with the CP group. In addition, rats pretreated with SPNPs showed a marked increase (15.9% and 38%) in GSH content in comparison with the Tel and free SP groups, respectively (*p* < 0.05).

#### 2.2.4. Effects of the Free SP Extract and the Nanoformulation on the PPAR-γ, Nrf-2, and HO-1 Signaling Pathways in Ovarian Tissues

**Figure 11:**
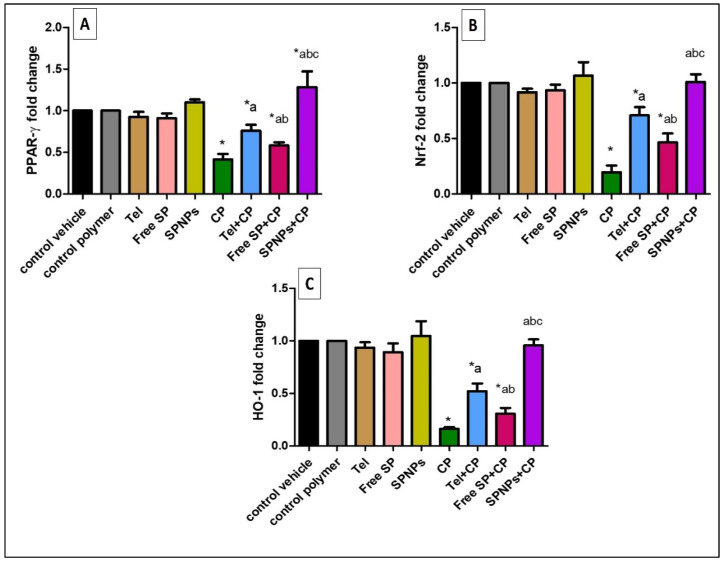
Effects of the free SP extract and the nanoformulation on the PPAR-γ, Nrf-2, and HO-1 signaling pathways in ovarian tissues. (**A**) PPAR-γ expression; (**B**) Nrf2 expression; (**C**) HO-1 expression. Data are presented as mean ± SD (n = 6). Each group differed significantly from the others at a *p*-value ≤ 0.05. * versus the control group, ^a^ versus the CP group, ^b^ versus the Tel+CP group, and ^c^ versus the Free SP+CP group. CP: Cyclophosphamide, SP: *Spirulina platenesis* extract, SPNPs: *Spirulina platenesis* extract nanoformulation.

As shown in Figure 11, the cyclophosphamide group showed a considerable downregulation (58.37%, 80.35%, and 83.7%, respectively) of the ovarian levels of PPAR-γ, Nrf-2, and HO-1 expression relative to the control group. On the other hand, pretreatment of rats with Tel, free SP extract, and SPNPs resulted in a substantial upregulation (82.45%, 39.42%, and 207.89%) of the mRNA expression of PPAR-γ, a marked upregulation (262.2%, 134.69%, and 415.3%) of the mRNA expression of Nrf-2, and a substantial upregulation (219.63%, 88.34%, and 486.5%) of the mRNA expression of HO-1 relative to the CP group. Moreover, the rats pretreated with SPNPs showed a remarkable upregulation (69.3%, 42.25%, and 83.49%, respectively) of the ovarian levels of PPAR-γ, Nrf-2, and HO-1 expression in comparison with the Tel group and a considerable upregulation (120%, 117.67%, and 211.4%, respectively) relative to the free SP group (*p* < 0.05).

#### 2.2.5. Effects of Free and Nanoformulation of SP on CP-Induced Ovarian Histopathological Changes

**Figure 12:**
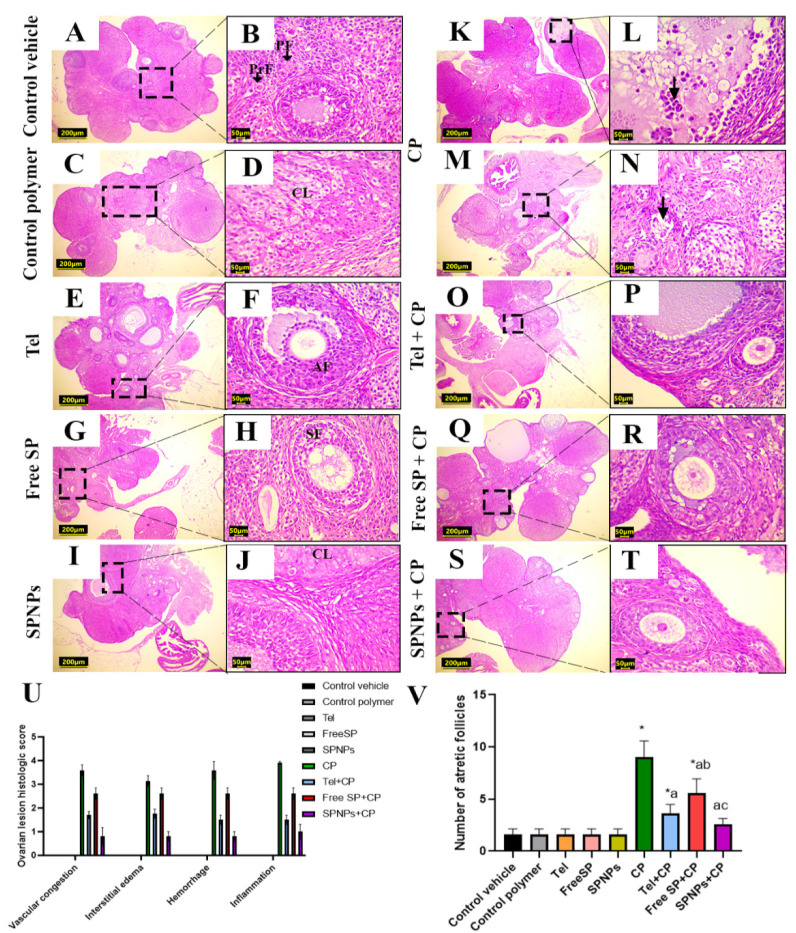
Representative light photomicrographs of rats’ ovarian tissue stained with hematoxylin and eosin (H and E). (**A**,**B**) The control vehicle, (**C**,**D**) control polymer, (**E**,**F**) telmisartan (Tel), (**G**,**H**) free *Spirulina platensis* extract (FreeSP), and (**I**,**J**) nanoformulation of *Spirulina platensis* extract (SPNPs) showed normal ovarian histoarchitecture and follicular developmental stages, including the primordial follicle (PrF), primary follicle (PF), secondary follicle (SF), and antral follicle (AF). (**K**–**N**) The cyclophosphamide (CP)-intoxicated group showed marked vascular congestion and multiple atretic follicles with the sloughing of granulosa cells into the lumen (arrows). (**O**,**P**) Tel+CP. (**Q**,**R**) Free SP+CP. (**S**,**T**) SPNPs+CP. (**U**) Ovarian lesion histological score; (**V**) number of atretic follicles. Scale bar (**A**,**C**,**E**,**G**,**I**,**K**,**M**,**O**,**Q**,**S**): 200 µm. Scale bar (**B**,**D**,**F**,**H**,**J**,**L**,**N**,**P**,**R**,**T**): 50 µm. Data are presented as the mean ± SD (n = 6). Each group differed significantly from the others at a *p*-value ≤ 0.05. * versus the control group, ^a^ versus the CP group, ^b^ versus the Tel+CP group, and ^c^ versus the Free SP+CP group.

The histopathological analysis of ovarian tissue from the various experimental groups is presented in Figure 12. The groups treated with the control vehicle, control polymer, Tel, free SP extract, and SPNPs exhibited normal ovarian histoarchitecture with different stages of follicular development (Figure 12A–J). In contrast, administration of CP resulted in extensive ovarian damage characterized by marked vascular congestion, hemorrhage, and edema. The CP group showed a lack of follicular stages of development, multiple atretic follicles, and interstitial mononuclear inflammatory infiltrations (Figure 12K–N). The free SP+CP group exhibited similar lesions to those in the CP toxic group but with less severity and a smaller distribution (Figure 12Q,R). The Tel+CP (Figure 12O,P) and SPNPs+CP (Figure 12S,T) groups showed nearly normal histoarchitecture with mild congestion of vessels, hemorrhage, and mononuclear cell infiltration, along with the restoration of follicular developmental stages.

The semiquantitative lesion scoring did not reveal significant changes in the groups treated with the control vehicle, control polymer, Tel, free SP extract, and SPNPs. However, the CP-treated group exhibited considerable increases in the ovarian histological score and the number of atretic follicles compared with those in the control rats. Furthermore, pretreatment of CP-injected rats with Tel, free SP extract, and SPNPs demonstrated substantial declines in the ovarian histological score and the number of atretic follicles compared with those of the control rats (Figure 12U,V).

#### 2.2.6. Effects of the Free SP Extract and the Nanoformulation on the Immunohistochemical Expression of PPAR-γ/NF-κB/TNF-α/Caspase-3 in CP-Induced Ovarian Damage

**Figure 13:**
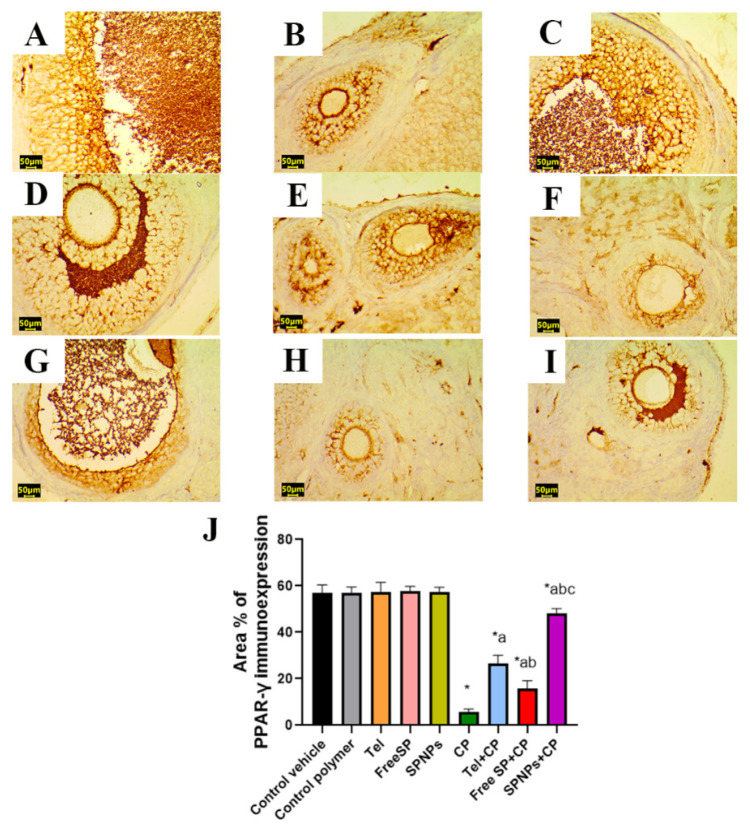
Representative light photomicrographs of rats’ ovarian tissue immunostained with peroxisome-proliferator-activated receptor gamma (PPAR-γ). (**A**) The control vehicle, (**B**) control polymer, (**C**) telmisertan (Tel), (**D**) free *Spirulina platensis* extract (Free SP), (**E**) nanoformulation of *Spirulina platensis* extract (SPNPs) showed marked PPAR-γ immunoexpression. (**F**) cyclophosphamide (CP) showed mild PPAR-γ immunoexpression. (**G**) Tel+CP showed moderate to marked PPAR-γ immunoexpression. (**H**) Free SP+CP showed moderate PPAR-γ immunoexpression, and (**I**) SPNPs+CP showed intense PPAR-γ immunoexpression. (**J**) The area % of PPAR-γ immunoexpression. Scale bar: 50 µm. Data presented as the mean ± SD (n = 6). Each group differed significantly from the others at a *p*-value ≤ 0.05. * versus the control group, ^a^ versus the CP group, ^b^ versus the Tel+CP group, and ^c^ versus the Free SP+CP group.

As shown in Figure 13, no significant differences were detected in the PPAR-γ immunoreactivity levels among the groups treated with the control vehicle, control polymer, Tel, free SP extract, and SPNPs. In contrast, the CP group showed a significant downregulation (77.38%) of PPAR-γ immunoreactivity relative to the control group. In contrast, rats pretreated with Tel, free SP extract, and SPNPs showed a significant upregulation (354%, 165%, and 721%) of the immunoexpression of PPAR-γ in comparison with the CP group. Moreover, rats pretreated with SPNPs showed a significant upregulation (80.8%) of the ovarian levels of PPAR-γ in comparison with the Tel group and a significant upregulation (209%) relative to the free SP group.

**Figure 14:**
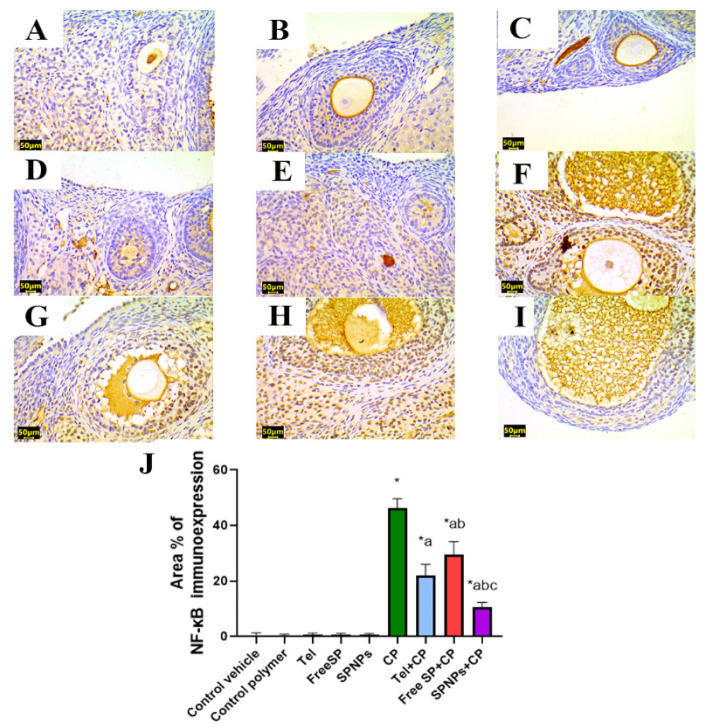
Representative light photomicrographs of rats’ ovarian tissue immunostained with nuclear factor kappa B (NF-κB). (**A**) The control vehicle, (**B**) control polymer, (**C**) telmisertan (Tel), (**D**) free *Spirulina platensis* extract (Free SP), and (**E**) nanoformulation of *Spirulina platensis* extract (SPNPs) showed negative NF-κB immunoexpression. (**F**) Cyclophosphamide (CP) showed intense NF-κB immunoexpression. (**G**) Tel+CP showed mild NF-κB immunoexpression. (**H**) Free SP+CP showed moderate NF-κB immunoexpression. (**I**) SPNPs+CP showed weak NF-κB immunoexpression. (**J**) The area % of NF-κB immunoexpression. Scale bar: 50 µm. Data are presented as the mean ± SD (n = 6). Each group differed significantly from the others at a *p*-value ≤ 0.05. * versus the control group, ^a^ versus the CP group, ^b^ versus the Tel+CP group, and ^c^ versus the Free SP+CP group.

**Figure 15:**
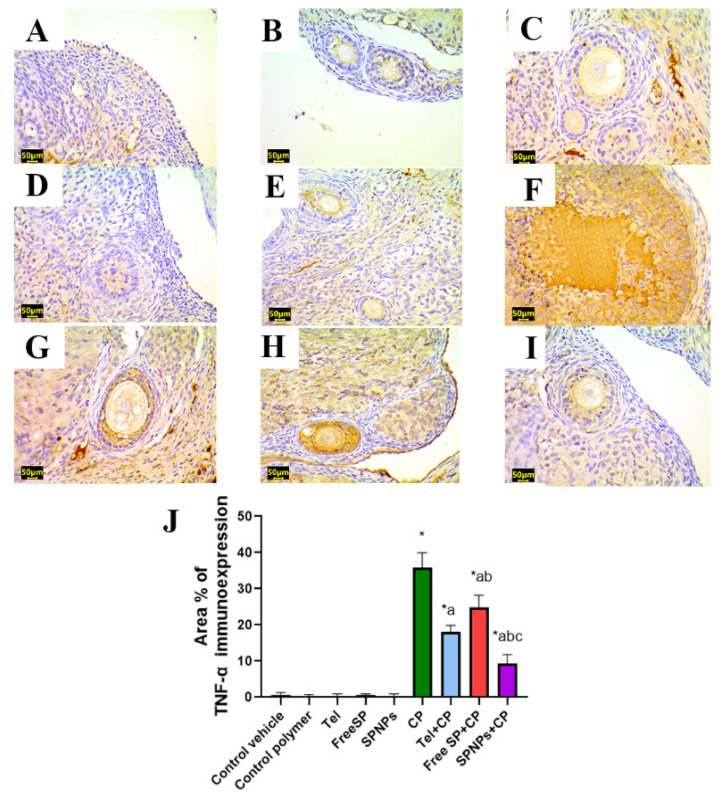
Representative light photomicrographs of rats’ ovarian tissue immunostained with tumor necrosis factor-alpha (TNF-α). (**A**) The control vehicle, (**B**) control polymer, (**C**) telmisertan (Tel), (**D**) free *Spirulina platensis* extract (Free SP), and (**E**) nanoformulation of the *Spirulina platensis* extract (SPNPs) showed negative TNF-α immunoexpression. (**F**) Cyclophosphamide (CP) showed intense TNF-α immunoexpression. (**G**) Tel+CP showed mild TNF-α immunoexpression. (**H**) Free SP+CP showed moderate TNF-α immunoexpression. (**I**) SPNPs+CP showed weak TNF-α immunoexpression. (**J**) The area % of TNF-α immunoexpression. Scale bar: 50 µm. Data are presented as the mean ± SD (n = 6). Each group significantly differed from the others at a *p*-value ≤ 0.05. * versus the control group, ^a^ versus the CP group, ^b^ versus the Tel+CP group, and ^c^ versus the Free SP+CP group.

To detect the beneficial impacts of Tel, free SP extract, and SPNPs on the alleviation of CP-induced ovarian inflammation, an assessment was conducted on the protein immunoexpression levels of NF-κB and TNF-α, which are the hallmarks of inflammation. There was no significant variation in NF-κB and TNF-α immunoexpression among the groups treated with the control vehicle, control polymer, Tel, free SP extract, and SPNPs. Relative to the control group, rats treated with CP showed an increase in inflammatory signals, as evidenced by noteworthy increases in NF-κB and TNF-α immunoreactivity (7819% and 4967%, respectively) in ovarian tissues. However, the administration of Tel, free SP extract, and SPNPs to rats intoxicated with CP led to a downregulation of inflammatory signals. This was illustrated by a considerable downregulation in the expression levels of NF-κB (52.5%, 36.06%, and 77.2%, respectively) and TNF-α (49.7%, 30.6%, and 73.8%, respectively) relative to the group treated with CP alone. Moreover, rats pretreated with SPNPs showed a significant decline (52.4% and 47.94%, respectively) in the ovarian levels of NF-κB and TNF-α in comparison with Tel and a significant reduction (64.3% and 62.2%, respectively) relative to the free SP group (Figure 14 and Figure 15).

**Figure 16:**
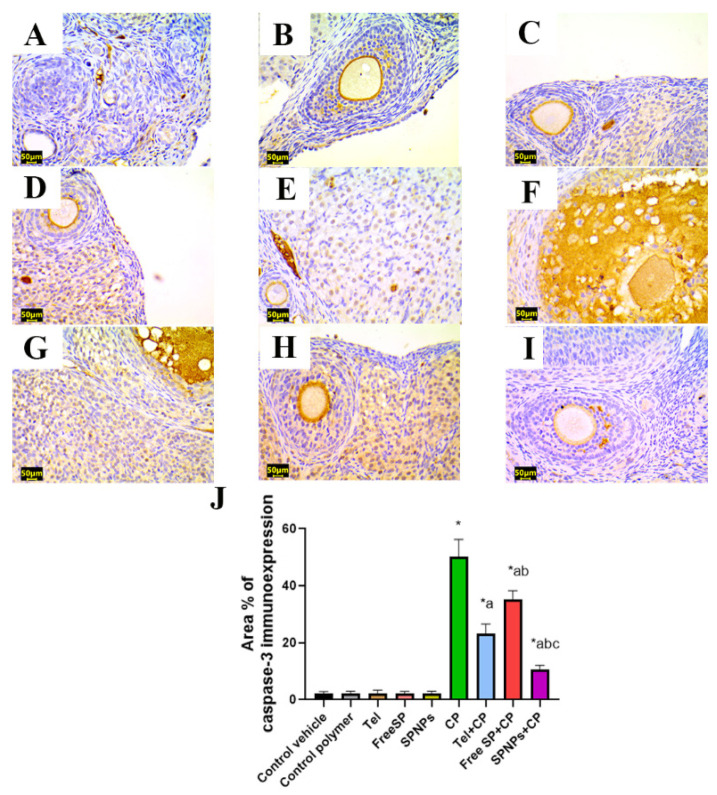
Representative light photomicrographs of rats’ ovarian tissue immunostained with caspase-3. (**A**) The control vehicle, (**B**) control polymer, (**C**) telmisertan (Tel), (**D**) free *Spirulina platensis* extract (Free SP), and (**E**) nanoformulation of the *Spirulina platensis* extract (SPNPs) showed mild caspase-3 immunoexpression. (**F**) Cyclophosphamide (CP) showed marked caspase-3 immunoexpression. (**G**) Tel+CP showed mild caspase-3 immunoexpression. (**H**) Free SP+CP showed moderate caspase-3 immunoexpression. (**I**) SPNPs+CP showed weak caspase-3 immunoexpression. (**J**) The area % of caspase-3 immunoexpression. Scale bar: 50 µm. Data are presented as the mean ± SD (n = 6). Each group significantly differed from the others at a *p*-value ≤ 0.05. *: versus the control group, ^a^ versus the CP group, ^b^ versus the Tel+CP group, and ^c^ versus the Free SP+CP group.

To determine the beneficial impacts of Tel, free SP extract, and SPNPs on the alleviation of CP-induced ovarian apoptosis, the protein immunoexpression level of caspase-3 was evaluated within the ovarian tissue, as caspase-3 serves as an indicator of apoptosis. There were no significant variations in caspase-3 immunoexpression among the groups treated with the control vehicle, control polymer, Tel, free SP extract, and SPNPs. Relative to the control group, rats treated with CP showed an increase in apoptotic signals, as evidenced by a noteworthy increase in caspase-3 immunoreactivity by 223% in ovarian tissues. Contrarily, the administration of Tel, free SP extract, and SPNPs to rats intoxicated with CP led to a downregulation of the apoptotic signal. This was illustrated by a considerable decrease in the expression of caspase-3, reaching 53.6%, 29.8%, and 78.8%, respectively, relative to the group treated with CP alone. Moreover, rats pretreated with SPNPs showed a significant downregulation (54.3%) of the ovarian levels of caspase-3 in comparison with Tel and a significant downregulation (69.8%) relative to the free SP group (Figure 16).

## 3. Discussion

The unicellular cyanobacterium *Spirulina platensis* (SP) has high nutritional value and a variety of therapeutic uses. *Spirulina* and its main ingredients—C-phycocyanin and phenolic and flavonoid compounds—have anti-inflammatory, neuroprotective, hepatoprotective, immunomodulatory, and anticancer properties, in addition to the ability to scavenge free radicals and provide antioxidant benefits [16,29]. *Spirulina* has garnered attention because of its perceived non-toxicity and substantial multiorgan protection against various drug- and chemical-induced toxic attacks [29]. The results showed that the SP ethanolic extract had high antioxidant activity and total contents of phenolic and flavonoid compounds. This result was in agreement with the findings of Scaglioni et al. [30], who observed that the high phenolic content of aqueous extracts of *Nannochloropsis* sp. and *Spirulina* sp. might have antioxidant properties. Furthermore, the total antioxidant activity, reducing power, and antioxidant activity when using DPPH showed a good association with the estimated amounts of phenol in the different extracts of SP. Scaglioni et al. [30] and Zaid et al. [31] verified the antioxidant properties of the phenolic compounds present in aqueous extracts of SP in a similar manner.

In a related context, the HPLC analysis of phenolic and flavonoid compounds in the SP ethanolic extract showed that there were many active compounds (catechol, syringic, rutin, luteolin, and quercetin) with various biological activities, such as antioxidant, anti-inflammatory, hepatoprotective, neuroprotective, nephroprotective, and anticancer activity [32,33,34,35,36]. 

Phenolic and flavonoid compounds exhibit antioxidative properties through various mechanisms. These include the scavenging of radical nitrogen species (RNSs) or radical oxygen species (ROSs), inhibition of free radical generation and upregulation of antioxidant defense systems. Phenolic and flavonoid compounds have antioxidant capacities at low contents, while at high contents they may exhibit pro-oxidant properties [37,38]. 

Furthermore, many bioactive compounds, such as hexadecanoic acid–methyl ester, 2,5-octadecadienoic acid, methyl ester, phytol, 3,7,11,15-tetramethyl-2-hexadecen-1-ol, and 9-octadecenoic acid (Z)-,2-hydroxy-1-(hydroxymethyl)ethyl ester were shown in the GC-MS analysis of the SP ethanolic extract. These compounds may have been the cause of the extract’s antioxidant action [33]. For example, hexadecanoic acid–methyl ester (palmitic acid) is a fatty acid that has antioxidant, antibacterial, and anti-inflammatory properties, as well as the ability to ameliorate the effects of different toxic agents [34]. In addition, it is a precursor to several compounds, including vitamins, phospholipids, glycolipids, prostaglandins, and prostacyclins [35].

Phytol (PYT) is a diterpene belonging to the long-chain unsaturated acyclic alcohol family. Numerous PYT studies have demonstrated anxiolytic, metabolism-modulating, antioxidant, antinociceptive, anti-inflammatory, immune-modulating, and antibacterial qualities [36].

The cyclic diterpene 3,7,11,15-tetramethyl-2-hexadecane-1-ol, which is sometimes referred to as phytol alcohol, is a component of the chlorophyll molecule. It has also been recognized to serve as a prophylactic for reactive oxygen species and a precursor for vitamin E and vitamin K1 [35]. The tested SP ethanolic extract included a large number of other chemicals with confirmed biological activities. These compounds may influence the protective effects of SP ethanolic extract in a synergetic manner.

In the same context, chitosan (Cs) is a natural polymer that is biodegradable and biocompatible and possesses mucoadhesive properties with a wide range of biological activities [37]. The crosslinking solution (NaTPP) was infused with a polysaccharide solution (chitosan) drop by drop at 4 °C. The crosslinking agents diffused into the internal structure of the polymer droplets from the continuous outer phase to form a bead matrix. The gel formed instantaneously, and the sol-gel transition process proceeded quickly in the newly formed hydrogel beads’ outer layer. The counter-ions continued to migrate through the layers into the particles’ core, generating a heterogeneous gelation profile with the ions and polymer functional groups interacting most at the surface and least at the core. The weight ratio of polymer to free SP extract to TPP was 3:1:0.1 [38]. This has been used to increase the absorption of beneficial compounds. It has been shown, for example, that combining tea polyphenols with Cs nanoparticles improves phenol absorption and bioavailability [39].

Because of its polycationic nature, Cs is water-soluble and has bioadhesive properties that allow it to rapidly adhere to negatively charged surfaces such as mucosal membranes. As a result, it increases adherence to the mucosa, thereby increasing the contact time for drug molecules to penetrate and exert their effects. Anionic drug delivery methods, including those involving low-molecular-weight medicines, can benefit from the complex properties of Cs. Its carrier ability increases with its charge, allowing it to function as a pH-dependent drug carrier [22].

The mechanism underlying Cs’s permeation-enhancing effect is also based on positive charges in the polymer that collaborate with the cell membrane and are principal in structural reorganization. It is also worth noting that Cs has efflux pump inhibitory characteristics. In this case, Cs inhibits specific transporter proteins on the membrane of enterocytes that pump out xenobiotics. They primarily include medicines. This nature makes these transporters a key component of drug resistance mechanisms [22].

The negative zeta potential can be attributed to the protein content of *Spirulina*, which is pH-dependent. The Alb and Glb protein fractions have isoelectric points close to 3.0 and 4.5, respectively, based on zeta-potential studies [40]. The Proprotein fraction, on the other hand, had a negative charge at pH levels ranging from 2 to 10. The modification of the ion gelation technique by employing temperature as a factor to enhance colloidal stability and uniform particle size and the utilization of an ice path allowed conservation at 4 °C in addition to colloidal protection during sonication [41].

SEM was also used to determine the nanostructure of the materials and the size range of SPNPs. The appearance of homogenous spherical particles with a diameter of less than 20 nm demonstrated that this was a successful technique utilizing low temperatures.

The DSC analysis of Cs showed an endothermic peak (78.33 C, −1.078 W/g), which could be attributed to the loss of water associated with hydrophilic groups. On the other hand, the exothermic peak (328.73 C, 7.85 W/g) could be explained by the decomposition of amine groups [42].

By comparing the DSC behavior of the free SP extract and SPNPs, it was clear that the endothermic peak of the free SP extract (87.08 C, −1.399 W/g) was shifted to a higher temperature (106.53 C, −1.677 W/g); moreover, the exothermic peak shifted to higher values. The exothermic peak of the free SP extract was at 303.1 C and 1.54 W/g, while the exothermic peak of the SPNPs was at 398.4 C and 4.13 W/g, and this appeared as a plateau rather than a characteristic peak. This can be explained by the protective effect of Cs, which surrounded the free SP extract (the ratio of Cs to free SP extract was 3:1). The results of the XRD analysis coincided with previous results that confirmed the size reduction and amorphous nature of the free SP extract, which could have been related to the greater content of hemicellulose than that of cellulose [43].

Therefore, this study aimed to determine the bioactive compounds in the SP ethanolic extract and use the ionic gelation method to synthesize the SP ethanolic extract/chitosan nanoparticles. Additionally, the study aimed to evaluate the protective effects of both the SP ethanolic extract and its nanoparticles against cyclophosphamide-induced ovarian toxicity, comparing them with an angiotensin II type 1 receptor antagonist (telmisartan).

Cyclophosphamide has a chemotherapeutic function by inhibiting cell division through the induction of DNA damage [44,45,46]. Cyclophosphamide metabolism creates alkylating chemicals, including 4-hydroxy cyclophosphamide, aldophosphamide, mustard, and acrolein. According to Kern and Kehrer [44], acrolein is a hazardous molecule that causes oxidative stress, which accelerates the rate of apoptosis. This metabolite interacts with proteins, membrane lipids, and DNA, among other cell macromolecules [45,46]. Chemotherapy and cyclophosphamide-based immunosuppressive therapy have been used much less frequently due to their propensity to cause infertility in young patients and their toxicity in host tissues, especially the ovary and testis [47,48,49].

According to the current investigation, cyclophosphamide (200 mg/kg, IP, once) caused increased follicular atresia, which, in turn, caused PFOF. These mechanisms include inflammation, lipid peroxidation, and ovarian follicle death. There is a difference between the production of free radicals and the antioxidant system as a result of CP. In this investigation, there was a noteworthy downregulation of Nrf2, PPAR-γ, and HO-1 gene expression and a decrease in the activities of antioxidant enzymes, such as SOD and GSH, in the ovaries’ generation of reactive oxygen species. These results align with those of Abdel-Raheem et al. [45] and Kabirian et al. [46].

Nrf2 is a transcription factor that controls how cells react to reactive oxygen species [50,51]. Keap1 is linked to Nrf2 and stimulates Nrf2 degradation [52]. Once antioxidant and detoxification enzymes are enabled, Nrf2 is activated, enters the nucleus, and binds to the ARE [53]. Heme oxygenase-1 (HO-1), glutathione peroxidase (GPx), glutathione-S-transferase (GST), NADPH: quinone oxidoreductase-1 (NQO1), and superoxide dismutase (SOD) are only a few of the many downstream enzymes that Nrf2 contains [54]. In response to oxidative stress and inflammation, HO-1 is generated, protecting tissues from damage [54].

In the same context, different research demonstrated the efficacy of pharmacological activators for two important antioxidative pathways used in combination. These pathways involve two nuclear transcription factors: peroxisome-proliferator-activated receptor-γ (PPAR-γ) and nuclear factor erythroid 2p45-related factor 2 (Nrf2) [55]. The general characteristics of Nrf2 and PPAR-γ pathways are associated with the improvement of pathological and drug-induced oxidative toxicity [55,56].

A notable increase in the MDA level revealed the effects of elevated ROSs, which were the product of the oxidation of membrane lipids by oxidants. Oxidative stress in granulosa cells results in the death of these cells, which induces ovarian follicle atresia [55]. Furthermore, according to Spears et al. [48], CP is known to stop mouse ovarian granulosa cell growth and division in vitro, causing the cells to decrease. The loss of numerous follicles following CP treatment was visible in the present histopathological examination and the immunohistochemical investigation of caspase-3. This is contemporaneous with oxidative-stress-induced follicular atresia—mostly in granulosa cells, leading to a decrease in the number of follicles and an increase in follicular atresia. There was suppression of apoptosis [49], as well as an increase in the blood levels of FSH and LH and a decline in the serum levels of AMH and E2, which was consistent with the findings in [49].

In the present study, pretreatment of rats with Tel, free SP extract, and SP SPNPs demonstrated a significant reduction in the adverse effects of oxidative stress and inflammation on ovarian tissue. Notably, SPNPs exhibited a particularly remarkable and greater protective effect than that of Tel and the free SP extract.

In addition, the current study demonstrated an increase in atretic follicles following CP treatment through histopathological analysis, PPAR-γ, and the immunohistochemical expression of caspase-3. This observation was consistent with the modified functionality of antioxidant enzymes, thereby indicating the occurrence of follicular atresia induced by oxidative stress. Parallel to these findings, previous studies showed that the administration of CP resulted in atresia of growing follicles, increased granulosa cell apoptosis, and inhibition of the conversion of primordial follicles into primary follicles. Additionally, the overexpression of NF-κB and subsequent production of pro-inflammatory cytokines—mainly TNF-α—induced by CP have the potential to initiate the activation of the caspase cascade, which is known to induce apoptosis in ovarian follicles, as previously shown in [49].

In addition, telmisartan, a class of medication known as an angiotensin II type 1 receptor antagonist, was documented as exhibiting partial agonistic properties towards PPAR-γ. It was shown that Tel inhibited NF-κB with subsequent attenuation of TNF-α-induced IL-6 expression via PPARγ. An experimental study showed that Tel completely restored cardiac histoarchitecture and ameliorated NF-κB via a canonical pathway in cyclophosphamide-induced myocardial injury. *Spirulina*, a type of blue-green algae, possesses remarkable properties as a potent antioxidant molecule and is widely recognized for its pronounced antioxidant and antiapoptotic characteristics. C-phycocyanin, a protein-bound chromophore observed in SP, hampers the process of oxalate-induced lipid peroxidation while simultaneously averting damage across multiple tissue types. Furthermore, the investigation of the protective properties of SP in reproductive glands, particularly in the context of ovarian toxicity, revealed promising results. SP was found to restore the histoarchitecture of the ovaries, as evidenced by a reduction in atretic follicles and an increase in the antioxidant status of the ovaries. An in vitro study showed that *Spirulina* extract exhibited a significant reduction in ROSs and RNSs [57]. Furthermore, it caused a decrease in the release of cytochrome C, an increase in mitochondrial membrane potential, and a reduction in the overexpression of the proapoptotic protein Bax in H9c2 cells [58].

## 4. Materials and Methods

### 4.1. Drugs and Chemicals 

Extra-pure chitosan (10–150 mPas, 90% DA (poly (D-glucosamine))) and deacetylated chitin (viscosity: 10–120 mPa.s (20 °C)) were obtained from Sisco Research Laboratory, India. Sodium tripolyphosphate (85%) was obtained from Lanxess Company, India. Acetic acid (96%) was obtained from Research-Lab Fine Chem Industries, India, and sodium hydroxide (NaOH), Tween 80, and glycerin with high purity were purchased from El Nasr Pharmaceutical Chemicals Co., Cairo, Egypt. Deionized water was obtained from Stakpure (Waters, Milford, Massachusetts, USA). Analytical-grade ethanol was bought from Merck (Darmstadt, Germany). Cyclophosphamide was obtained from Baxter Oncology GmbH CO., Halle, Germany. Telmisartan was obtained from Sigma-Aldrich, Saint Louis, USA. All other chemicals were utilized with the highest analytical grade and good quality.

### 4.2. In Vitro Investigation

#### 4.2.1. *Spirulina platensis* (SP) Cultivation

A modified type of Zarrouk’s medium was used to culture *Spirulina platensis* (SP) [59]. Axenic culture flasks were kept at 27 ± 3 °C with 45 µmol photon m^−2^ s^−1^ of light intensity. To harvest the cultures’ development, a combination of dry-filtered air (97%) and 3% CO_2_ was used.

The previous culture was extracted by centrifuging at 4000 rpm for 15 min (Sigma 2-16KL Centrifuge, up to 20,000× *g* with fixed-angle rotors with a max speed of 15,300 rpm). Following two washes [54], the cultures were dried in a freeze-dryer for twelve hours. They were kept at −20 °C until use.

#### 4.2.2. Preparation of the Ethanolic Extract of *Spirulina Platensis*

Lyophilized SP powder (10 g) was shaken (VS-8480) for 72 h at 25 °C after being added to 100 mL of ethanol (80%). The ethanolic extract was obtained by centrifuging at 4000 rpm for 15 min (Sigma 2-16KL Centrifuge, Osterode, Germany, up to 20,000× *g* with fixed-angle rotors and a max speed of 15.300 rpm).

The final freeze-dried ethanolic extract of SP powder was obtained by concentrating the collected supernatant after extraction in a rotary vacuum evaporator and freeze-drying it (yielding 20% of the initial amount of dry powder of *Spirulina platensis* biomass). It was then stored at 4 °C and shielded from light until it was utilized as directed by [55] with some modifications.

#### 4.2.3. Evaluation of the Antioxidant Activity of SP Ethanolic Extract Using the DPPH Radical Scavenging Method

The free radical scavenging activity of the SP ethanolic extract was estimated, as noted in [56]. A UV-visible spectrophotometer (UV-VIS Milton Roy SpectraLab Scientific Inc., Markham, ON, Canada) was used to measure the absorbance at 517 nm. Ascorbic acid was used as a reference compound for calibration. The absorbance values obtained were then utilized to construct a logarithmic dosage inhibition curve (n = 3), and the IC_50_ value was determined. The following formula was used to calculate the percentage of the DPPH scavenging effect:

(1) $$\mathrm{DPPH scavenging effect (\%) or percent inhibition}=\frac{A0-A1}{A0}\times 100$$

where A1 represents the absorbance when a test or standard sample was present, and A0 represents the absorbance of the control response.

#### 4.2.4. Total Phenolic Content (TPC) of the SP Ethanolic Extract

Using theFolin–Ciocalteu reagent and a colorimetric technique (UV-visible spectrophotometer (UV-VIS Milton Roy, SpectraLab Scientific Inc., Markham, Canada)) at 765 nm, the total phenolic content of the SP ethanolic extract was determined [56]. The experiment was performed in triplicate. The total phenolic content of the samples was calculated as equivalent to mg of gallic acid/g of dry weight (mg GAE/g DW).

#### 4.2.5. Total Flavonoid Content (TFC) of the SP Ethanolic Extract 

In a modified AlCl3 calorimetric method (UV-visible spectrophotometer (UV-VIS Milton Roy, SpectraLab Scientific Inc., Markham, ON, Canada )), the total flavonoid content of the SP ethanolic extract was detected at 510 [57]. The experiment was performed in triplicate. The flavonoid content of the extract was estimated by using the quercetin standard calibration curve, and the results obtained for the flavonoids were expressed as the equivalent to mg of quercetin (Qu)/g of dry wt (mg QuE/g DW).

#### 4.2.6. Analyses of Phenolic and Flavonoid Compounds in the SP Ethanolic Extract Using High-Performance Liquid Chromatography (HPLC)

With an HPLC system (Agilent Series 1100, Agilent, Frederick, CO, USA), phenolic and flavonoid component studies employing the SP ethanolic extract (10 mg dissolved in 1 mL of ethanol (80%) with 25 μL injection volumes) were carried out. The methods outlined by Lin et al. (1996) and Chang et al. (2002) were used, and the system consisted of an auto-sampling injector, solvent degasser, two LC-pumps (series 1100) with the ChemStation software 1100, and a UV-Vis detector (set to 250 nm for phenolic acids and 360 nm for flavonoids). A C18 column (125 mm × 4.60 mm, 5 µm particle size) was used for the study. Using a gradient mobile phase consisting of two solvents (Solvent A: methanol; Solvent B: acetic acid in water (1:25)), phenolic acids were isolated. Using a gradient mobile phase consisting of two solvents (Solvent A: methanol; Solvent B: acetic acid in water (1:25)), phenolic acids were isolated. Using an isocratic elution (70:30) procedure, flavonoids were separated using a mobile phase consisting of two solvents: (A) acetonitrile and (B) 0.2% (*v*/*v*) aqueous formic acid. There was a 1 mL/min flow rate for the solvent. The separation procedure was carried out at 25 °C. 

#### 4.2.7. Gas Chromatography-Mass Spectrometry (GC-MS) Analysis of the SP Ethanolic Extract

GC/MS equipment (Thermo Scientific, Lenexa USA, Trace GC-ISQ mass spectrometer) was utilized to analyze the chemical composition of the SP ethanolic extract (10 mg dissolved in 1.5 mL ethanol (80%) with 3 μL injection volumes). The temperature program was intended to go from 50 to 280 °C at a rate of 10 °C per minute. The injector was 220 °C, the interface was 220 °C, and the source temperature was 200 °C. El-Kareem et al. (2016) reported that helium was utilized as a carrier gas at a flow rate of 1 milliliter per minute. The components were tentatively identified by comparing the retention times of components and mass spectra with mass spectral databases (NIST 05 (NIST/EPA/NIH mass spectral library version 2.0d) and WILEY (Wiley Registry of Mass Spectral Data, 9th Edition Version 1.02)).

#### 4.2.8. Preparation of SP Nanoparticles (SPNPs) Using the Ionic Gelation Method 

Chitosan nanoparticles cross-linked using sodium tripolyphosphate (TPP) were prepared with a modified ionotropic gelation method [24]. Three grams of chitosan (Cs) were dispersed in 150 mL of 1% acetic acid using a magnetic stirrer (Stuart, Calibre Scientific, USA) at 200 and 50 °C for 30 min. One carefully weighed gram of free SP extract was triturated with a sufficient amount of glycerin (1 mL) to produce a smooth paste; then, 10 mL of 1% Tween solution was allowed to be sonicated (SONIC VIBRA CELL, USA) for 5 min in an ice bath (10 s of pulsing and 5 s of break) at 75% of its power (130 W). The homogenous, free SP extract dispersion was added stepwise to the chitosan solution. Then, it was stirred for an additional hour. The dispersion was allowed to be sonicated (SONIC VIBRA CELL, Newtown, CT, USA) for 5 min in an ice bath (10 s of pulsing and 5 s of break) at 75% of its power (130 W), and the pH was adjusted to 5 (Benchtop 3510 pH/MV, Jenway, Felsted, UK) with the aid of 4% NaoH. The dispersion was transferred to a pre-refrigerated amber bottle immersed in an ice bath (equilibration at −80 °C for 4 h, ultra-low-temperature freezers, BINDER, Pforzheim, Germany), allowing the dispersion to equilibrate the temperature at 4 °C. Then, 4 mL of TPP 2.5% (*w*/*v*) was added stepwise with the aid of a 20 mL syringe, and stirring was continued for an additional 30 min. Finally, the nanoparticles were separated with the aid of a cooling centrifuge at 10,000 rpm for 10 min (Centrifuge benchtop Centurion Scientific Pro Research K241R, Wolflabs, Pocklington, United Kingdom, up to 20,000× *g* with fixed-angle rotors and a max speed of 10,000 rpm) at −4 °C. Then, the resulting nanoparticles were washed twice with deionized water and allowed to freeze until completely dry (Christ Benchtop Freeze Dryer, Osterode am Harz, Germany). Chitosan nanoparticles were prepared using the same procedures except for the addition of SP powder.

#### 4.2.9. Characterization of SP Nanoparticles

##### The Percentage of Coacervation Yield

The percentage yield was calculated to analyze the efficiency of the manufacturing operations. The total amount of powder was measured using an analytical weighing scale (Sartorious, New York, NY, USA) after lyophilization, and the percentage yield was determined using the formula below

(2) $$\mathrm{\% yield}\frac{total\;amount\;of\;SPNPs}{total\;amount\;of\;all\;ingredients\;(\;STPP+chitosan+FreeSP)}\times 100$$

##### Average Particle Size and Zeta-Potential Evaluation

The particle size of SPNPs determines their effectiveness, whereas the zeta potential is an indicator of their colloidal stability [60]. They were measured using the Zeta Sizer Nano (Malvern Panalytical Ltd., Malvern, UK).

##### Transmission Electron Microscopy (TEM)

The nanoparticles were suspended in ethyl alcohol before being put on a carbon grid and air-dried. A transmission electron microscope (TEM, JEM2100F electron microscope, JEOL, Ltd., Tokyo, Japan) was used to examine and photograph the material. 

##### Thermal Stability (DSC)

Differential scanning calorimetry (TA instruments, Waters LIC, Milford, CT, USA) was performed to examine the thermal behavior of the samples of free SP extract, polymer (Cs), and SPNPs. Each sample was carefully weighed (2–4 mg) using a microbalance (Sartorius, Göttingen, Germany), and the samples were heated from 0 to 400 °C (10 °C/min).

##### X-ray Diffraction Analysis (XRD)

SPNPs and free SP extract were subjected to XRD analysis. The X-ray diffractograms were acquired based on Bragg’s law using an XRD diffractometer (APD2000 pro, GNR, Italy, CRYSTAL IMPACT software, Bonn, Germany) with CuK radiation, a monochromatic voltage of 35 kV, and an electric current of 25 mA. The 2ɵ diffraction angle ranged from 4.95° to 79.75°.

### 4.3. In Vivo Investigation

#### 4.3.1. Study Animals

Adult female Wister albino rats weighing 160–180 g (12–16 weeks old) were obtained from a national research center in Egypt. The animals were fed a standard diet and water ad libitum. The research adhered to institutional guidelines and ethical requirements for the proper treatment of animals. The study protocol had been approved by a research ethics committee, Faculty of Pharmacy, University of Tanta, which has adhered to the ARRIVE 2.0 guidelines (Code of Protocol: TP/RE/12/23p-062) and also from Princess Nourah bint Abdulrahman University, with IRB registration number: HA-01-R-104.

#### 4.3.2. Experimental Design

Following seven days of acclimation, rats were split into nine groups, with each consisting of six rats, as shown in Table 7. 

#### 4.3.3. Sample Collection

On day 21 of the study, the rats were sacrificed 48 h after receiving cyclophosphamide (CP). The rats were given isoflurane anesthesia; then, the blood was withdrawn through a cardiac puncture and collected in clean, dry test tubes. After that, the animals were cervically dislocated and killed. The tubes were allowed to stand for 15 min to clot at 4 °C. Blood samples were collected and subjected to centrifugation at 4000 rpm for 15 min (Sigma 2-16KL Centrifuge, Osterode, Germany, up to 20,000× *g* with fixed-angle rotors with a max speed of 15,300 rpm) at 4 °C to obtain serum for hormone analysis. Both ovaries were carefully harvested and preserved in 10% buffered formalin solution at room temperature for subsequent histopathological and immunohistochemical assessments. Additional ovarian tissue samples were stored at −80 °C for biochemical investigations.

#### 4.3.4. Biochemical Analysis 

Serum follicle-stimulating hormone (FSH), luteinizing hormone (LH), and estradiol (E2) levels were quantitatively evaluated according to the manufacturer’s instructions for ELISA kits obtained from CUSABIO.co (cat. nos. CSB-E06869r, CSB-E12654r, and CSB-E05110r, respectively). In addition, anti-Mullerian hormone (AMH) and progesterone levels were investigated using ELISA kits obtained from AssayGenie.co and BioVendor.co (cat. nos. RTFI01398 and RTC008R, respectively). 

#### 4.3.5. Investigation of Oxidative Stress Biomarkers 

Reduced glutathione (GSH) and lipid peroxidation (MDA) contents were measured in ovarian tissues using various commercial ELISA kits purchased from MyBioSource.co., San Diego, CA, USA and CUSABIO.co., Houston, TX, USA (cat. nos. MBS268427 and CSB-E12144r, respectively). The assay procedures were performed according to the manufacturer’s instructions. 

#### 4.3.6. Real-Time Quantitative (qRT-PCR) Analysis of PPAR-γ, Nrf2, and HO-1

Total RNA was extracted using the easy-spinTM total RNA extraction kit (iNtRON Bio, Inc., Seoul, Korea). A quantiTect reverse transcription kit was obtained from Bio Basic, Inc. (Markham, ON, Canada) (QIAGEN, Hilden, Germany). Melting curve analysis was performed at temperatures between 50 and 99 °C. The first step was held at 95 °C for 10 s, followed by 15 s of annealing at 60 °C; this was repeated 55 times.

Sequences of primers used are listed in Table 8. Using the 2^−ΔΔCt^ method, the relative mRNA expression of target genes was determined for the fold changes by normalizing the control group and the β-actin level [66].

#### 4.3.7. Histopathological Examination

Ovarian tissue specimens were carefully excised and immediately soaked in a 10% neutral buffered formalin solution. The specimens underwent a conventional method of paraffin embedding for tissue processing, followed by sectioning to a thickness of 4 μm. Subsequently, the specimens were stained using the hematoxylin and eosin (H and E) stain for pathological examination [71].

A morphometric evaluation was conducted to assess the extent of ovarian injury. This evaluation included a semiquantitative analysis of various pathological changes such as vascular congestion, interstitial edema, hemorrhage, and inflammation. Briefly, slides are assigned in random codes, and their order is randomized using Excel to be unaware of the treatment groups. A total of five fields were chosen at random from each slide to analyze the lesions in each sample. The severity of these lesions was classified using a 4-point scale based on the proportion of the affected region compared to the entire section [72,73] as follows: 0 = normal; 1 = 1–25%; 2 = 26–50%; 3 = 51–75%; 4 = 76–100%. Additionally, the technique of atretic follicle counting was utilized to determine the extent of follicular damage using Toup view software of a Toup cam (10MB, LCMos10000KPA, Hangzhou, China) [74,75].

#### 4.3.8. Immunohistochemical Detection of PPAR-γ, caspase-3, NF-kB, and TNF-α in Ovarian Tissue

The antioxidant status, apoptosis, and inflammation were evaluated through immunostaining for peroxisome proliferator-activated receptor gamma (PPAR-γ), caspase-3, nuclear factor kappa B (NF-κB), and tumor necrosis factor-alpha (TNF-α). Sections measuring 5 μm in thickness were obtained from each paraffin block through the utilization of a microtome. These sections were then mounted in positively charged slides. After deparaffinization, rehydration, retrieval of antigen, and the application of a blocking step to inhibit nonspecific binding of immunological reagents for immunohistochemical analysis, mouse monoclonal antibodies against PPAR-γ (Catalog # (E-8): sc-7273, 1:50 dilution, Santa Cruz), caspase-3 (Catalog # (E-8): sc-7272, 1:50 dilution, Santa Cruz), NF-κB (Catalog # (F-6): sc-8008, 1:50 dilution, Santa Cruz) and TNF-α (Catalog # (52B83): sc-52746, 1:50 dilution, Santa Cruz) were employed with an overnight incubation at 4 °C, followed by treatment with horseradish peroxidase enzyme conjugation for 20 min. Subsequently, the samples were exposed to 3,3′-diaminobenzidine tetrahydrochloride (DAB)-H_2_O_2_ for two min and then subjected to a washing step. Then Mayer’s hematoxylin was applied for counterstaining, and slides were mounted with DPX [76]. The antioxidant status, apoptosis, and inflammation were evaluated through immunostaining for peroxisome proliferator-activated receptor gamma (PPAR-γ), caspase-3, nuclear factor kappa B (NF-κB), and tumor necrosis factor-alpha (TNF-α). Sections measuring 5 μm in thickness were obtained from each paraffin block through the utilization of a microtome. These sections were then mounted in positively charged slides. Afterward, the samples were treated with 3,3′-diaminobenzidine tetrahydrochloride (DAB)-H_2_O_2_ for two minutes and then subjected to a washing step. Mayer’s hematoxylin was used for counterstaining, and the slides were mounted with DPX [76]. The slides were examined under a microscope, and the area percentage of the immunopositive reaction was determined by analyzing ten randomly selected fields from each rat within each group. These calculations were performed using the ImageJ analysis software 1.54 [77].

### 4.4. Statistical Analysis

Data had been expressed as the mean values ± standard deviation (mean ± S.D). The differences between groups were determined with one-way ANOVA followed by Tukey’s multiple- comparisons tests. Probability values (P) less than 0.05 were considered statistically significant. GraphPad Prism, version 5 (GraphPad Software Inc., La Jolla, CA, USA) was used for statistical calculations.

## 5. Conclusions

The current study suggests that a nanoformulation containing an ethanolic SP extract and chitosan obtained through the ionic gelation method can improve CP-induced ovarian damage by upregulating Nrf-2/PPAR-γ/HO-1 expression, resulting in anti-inflammatory and antioxidant properties, as well as the prevention of ovarian apoptosis.

## Figures and Tables

**Table 1:** Antioxidant, total phenolic, and total flavonoid contents of the SP ethanolic extract *.

Assay	SP Ethanolic Extract
DPPH (Inhibition %)	79.7 ± 1.3
Total phenolic content (mg GAE/g DW)	35.9 ± 0.5
Total flavonoid content (mg GAE/g DW)	81.17 ± 2.5

* Data are presented as the mean ± SD (n = 3).

**Table 2:** HPLC analysis of the phenolic compounds that occurred in the SP ethanolic extract *.

Paek No.	RT	Compounds	Concentration µg/mL
1	4.1	Catechol	10.31 ± 0.72
2	5.1	Syringgenic	12.89 ± 0.83
3	7.0	Cinnamic acid	3.78 ± 0.22
4	8.0	Caffeic acid	1.41 ± 0.02
5	9.4	Pyrogallol	1.39 ± 0.03
6	12.7	Unknown	--
7	15.0	Unknown	--

* Data are presented as the mean ± SD (n = 3).

**Table 3:** HPLC analysis of the flavonoid compounds that occurred in the SP ethanolic extract *.

Peak No.	RT	Compounds	Concentration µg/mL
1	4.6	Naringin	3.12
2	5.2	Rutin	6.20
3	7.0	Quersestin	15.30
4	8.1	Kampferol	3.51
5	9.0	Luteolin	6.41
6	10.0	Apegenin	3.36
7	12.01	Catechin	5.26
8	14.0	Unknown	--

* Data are presented as the mean ± SD (n = 3).

**Table 4:** GC-MS analysis of the SP ethanolic extract *.

RT	Compound Name	PA %	MF	Biological Activity
21.31	Heptadecane	2.83	C_17_H_36_	Antioxidant and antimicrobial activity
26.39	Hexadecanoic acid	8.08	C_16_H_32_O_2_	Antioxidant, antibacterial, and anti-inflammatory activity
29.10	Phytol	8.67	C_20_H_40_O	Anti-inflammatory, antioxidant, and antimicrobial activity
29.39	9,12-Octadecadienoic acid (Z, Z)	9.11	C_18_H_32_O_2_	Antioxidant, anticancer, and anti-inflammatory activity
29.54	9,12-Octadecadienoyl chloride (Z, Z)	9.45	C_18_H_31_CIO	Antibacterial and antifungal activity
30.03	Oleic acid	1.67	C_18_H_34_O_2_	Antioxidant, antibacterial, and anti-inflammatory activity
35.31	Hexadecanoic acid, 2,3-di-hydroxypropyl ester	1.89	C_19_H_38_O_4_	Antioxidant activity
37.95	9-Octadecenoic acid (Z,Z)-, 2-hydroxy-1-(hydroxymethyl)ethyl ester	5.82	C_21_H_38_O_4_	Antioxidant, anti-inflammatory, antimicrobial, and diuretic activity
42.73	Hexadecanoic acid, octadecyl ester	47.92	C_17_H_34_O_2_	Antioxidant and anti-inflammatory activity

* RT: retention time; PA: peak area; MF: molecular formula. The biological activities of different compounds were derived from the PubChem database website (https://pubchem.ncbi.nlm.nih.gov/).

**Table 5:** Characteristics of the polymeric SPNPs.

Parameter	Mean	Range
Percentage yield (%)	20.96 ± 2.89	18.29 to 24.3
Particle size (nm)	138.2 ± 7.18	132.6 to 146.3
Zeta potential (mV)	−28.33 to −33.69	−31.58 ± 2.86

**Table 6:** Results of the DSC analysis of free SP particles, a polymer (CS), and SPNPs.

	Endothermic Peak		Exothermic Peak	
	Peak (°C)	Heat Flow (W/g)	Peak (°C)	Heat Flow (W/g)
Free SP	87.08 °C	−1.399	303.1 °C	1.54
Polymer (Cs)	78.33	−1.078	328.73	7.85
SPNPs	106.53	−1.677	398.4	4.13

**Table 7:** Experimental protocol for evaluating the effects of free SP extract and the nanoformulation on cyclophosphamide-induced ovarian toxicity.

Group/n = 6	Dosage Protocol
1: Control vehicle	Rats received a daily oral dose of saline for 21 days.
2: Control polymer	Rats received daily oral doses of chitosan polymer (equivalent dose of 100 mg/kg, P.O. SPNPs for 21 days [61,62].
3: Telmisartan (Tel)	Rats received daily oral doses of telmisartan (10 mg/kg, P.O.) for 21 days [63].
4: *Spirulina platensis* extract (Free SP)	Rats received daily oral doses of *Spirulina platensis* extract (300 mg/kg, P.O.) for 21 days [64].
5: Nanoformulation of the *Spirulina platensis* extract (SPNPs)	Rats received daily oral doses of the nanoformulation of the *Spirulina platensis* extract (100 mg/kg, P.O.) for 21 days [17].
6: Cyclophosphamide (CP)	Rats received a daily oral dose of saline for 21 days with a single injection of CP (200 mg/kg. I.P.) on day 19 [65].
7: Tel+CP	Rats were treated with Tel (10 mg/kg. P.O.) for 21 consecutive days, and on day 19, a single dose of CP (200 mg/kg, I.P.) was given.
8: Free SP+CP	Rats were treated with free SP extract (300 mg/kg. P.O.) for 21 consecutive days, and on day 19, a single dose of CP (200 mg/kg, I.P.) was given.
9: SPNPs+CP	Rats were treated with SPNPs (100 mg/kg. P.O.) for 21 consecutive days, and on day 19, a single dose of CP (200 mg/kg, I.P.) was given.

**Table 8:** The sequence of primers for the studied genes.

Genes	Sequence (5′-3′)	References
PPAR-γ	F:CATTTCTGCTCCACACTATGAA R: CGGGAAGGACTTTATGTATGCG	[67]
Nrf2	F:CACATCCAGACAGACACCAGT R: CTACAAATGGGAATGTCTCTGC	[68]
HO-1	F: GGCTTTAAGCTGGTGATGGC R: GGGTTCTGCTTGTTTCGCTC	[69]
Β-actin	F: TCCTCCTGAGCGCAAGTACTCT R: GCTCAGTAACAGTCCGCCTAGAA	[70]

## Data Availability

Data is contained within the article.
